# What Fillings Should We Use

**Published:** 1885-10

**Authors:** W. G. A. Bonwill

**Affiliations:** Philadelphia.


ARTICLE VI.

### WHAT FILLINGS SHOULD WE USE?

DR. W. G. A. BONWILL, PHILADELPHIA.

When I look back at my commencement and reflect that my early practice was founded on what the older men in authority had published and taught, and how I feared to do other than they demanded, I shudder at the many teeth I extracted I now know might have been saved, with even the amalgam of that day. And I tremble at the advice *now* given by the authorities that *gold* only should be used as a permanent filling. Young men knew no better, but the older do. God forgive them, I cannot. While I do not belong to the disciples of the new departure, *so far as their theory is concerned*, I stand side by side with any person *who can save teeth by plastic materials*, where gold cannot be used. Better do this than persist with gold indiscriminately, and lose teeth, rather than stoop to conquer with *any article* that is *not gold.* The public are demoralized on the subject of *gold.* "Are you not going to fill my teeth with gold?" says nearly every new customer; "Dr. —— would not think of using anything else." A city operator must have more than the usual quota of courage to stand before the societies and state "he has been using *amalgam* more freely of late." For the first eight years of my practice I would not touch it, because Doctors Elisha Townsend and

J. D. White passed their anathemas on everything but gold and tin. I worked myself nearly to death with tin to find it preserves from caries but not from attrition. Since 1862, I have been feeling my way, and while I think I have reared many beautiful and substantial monuments of gold, and have perfected machinery with which to do it, yet I consume more amalgam than ever before.

A gold filling *properly* impacted, with cavity judiciously prepared, and the walls shaped as to forbid future decay, *will save*, irrespective of the frailty of their bony structure? But as thousands of teeth *cannot be so prepared*, both of strong and of frail organizations, and the circumstances *cannot* be controlled, we should resort to something that will enable us the more surely to meet the issue.

To enumerate the many cases of peculiar character that forbid the use of gold, would be too great a task. Physical impossibilities lie in the way of every undertaking; and it is for the successful engineer, who is well acquainted with his material, and their relative strength and *adaptability* for his purposes, to so use each, that his design will be consummated, and which shall not by future wear, prove a failure. There is a fitness in every material that experience has proven to be specially adapted for a given work, and when this general law is recognized and we become first-class engineers, we shall the better see where we can adapt our materials to the work to be done, and we can be the more certain of success, for it is founded on the logic of mechanics and physical law.

Where is the dentist that first lays out his design and orders materials best adapted for specific portions of it?

As well say everything should be made only of iron, or steel, or wood, as that every tooth should be filled with gold; or, as *equally ridiculous*, that the amalgam or some one of the plastic fillings should be the only material used.

It is not *necessary* to found a *creed or departure* on a law of *incompatibility* to tooth substance. We need not look so far into the unknown and unknowable. We poor, short-sighted creatures must have the tangible; not a hypothesis on a *supposed theory*. Any one with half an eye can see just where the incompatibility is; not between gold and dentos, but between dentos and untutored and unskilled brain and hands to *carry out the law of adaptibility*—the correlation of forces involved.

One skilled in the use of the mallet, with the rubber-dam and a substantial starting point, with walls ever so frail, can perfectly impact and complete the work in gold filling, *provided the surroundings are there.* But allow *one little vacuum* between the tooth substance and the filling, and a *capillary tube* will be formed to suck up *fermentable material*; and the *acid generated* will act on the tooth whether it be filled with gold, amalgam, oxyphosphate, or gutta percha. A thousand capillary tubes making porosity in the gold or the amalgam, will not do it; but if there is one, however small, between dentos and filling, destruction is sure.—*Transactions of the Odontological Society of Pennsylvania.*

